# Editorial: Photocatalytic functionalization of inert or unsaturated bonds

**DOI:** 10.3389/fchem.2024.1372572

**Published:** 2024-02-07

**Authors:** Yong Luo, Gu Zhan, Xuefeng Cong, Hang Zhang

**Affiliations:** ^1^ School of Pharmaceutical Sciences (Shenzhen), Sun Yat-sen University, Shenzhen, China; ^2^ State Key Laboratory of Southwestern Chinese Medicine Resources, School of Pharmacy, Chengdu University of Traditional Chinese Medicine, Chengdu, Sichuan, China; ^3^ Institute of Molecular Plus, Tianjin University, Tianjin, China; ^4^ School of Pharmaceutical Sciences, Zhengzhou University, Zhengzhou, Henan, China

**Keywords:** photocatalysis, unsaturated-bond-functionalization, inert-bond-activation, mechanistic study, radical process, synthetic application

Photoredox catalysis has continued to expand at a rapid pace in the last few decade, both in terms of the diversity of the transformations and the abundance of the catalytic scenarios. By utilizing simple and mild reaction conditions, photocatalysis provides an intuitive strategy to construct valuable molecules in a green fashion. In this field, the functionalization of unsaturated and inert bonds represents one of the most powerful synthetic tools to build up architecturally complex molecules. In this context, we have announced a Frontiers Research Topic, entitled *Photocatalytic functionalization of inert or unsaturated bonds,* to collect recent achievements in visible-light-induced transformations of inert, double, or triple bonds. Contributions to this Research Topic include explorations of photoredox functionalization of sp^2^ and sp^3^ C-H bonds, difunctionalization of C=C bonds, and the photophysical properties of unsaturated compounds, outlining the reaction design, mechanism study, and synthetic application in photochemistry.

In recent years, several reviews (Wang et al., 2018; Holmberg-Douglas and Nicewicz, 2021; Bellotti et al., 2023) have provided good summaries on the utilization of photoredox catalysis in C-H bond functionalization and obtained high citations, highlighting the great interest of chemical scientists in this field. These reviews suggested that one of the strategies to cleave the C-H bond is the generation of a carbon-centered radical via homolysis, and the other is the liberation of a proton after reacting with other reactive species. By using the latter strategy, Li et al. reported a simple synthetic route for the sulfonylation of the C-H bond in anilines from sulfonyl fluoride. The cross-coupling of the sulfonyl radical generated from visible light-mediated S-F bond cleavage and the carbon-centered radical resulting from the oxidation of aniline and subsequent deprotonation gave rise to various diaryl sulfones in moderate to good yields. Due to their good stability under various modification reaction conditions, sulfonyl fluorides allow late stage functionalization to generate complex products unattainable by traditional methods. In addition, the ion-pair effect was observed to have a crucial influence on efficiency, as the iridium complex bearing the chloride anion exhibited a superior advantage over the hexafluorophosphate anion.

In terms of sp^3^ C-H bond functionalization, 1,5-hydrogen atom transfer (HAT) has been demonstrated to be an efficient protocol to generate alkyl radicals and proceed to further transformations. Tu et al. described a photocatalytic chemodivergent approach to 1-pyrrolines and 1-tetralones via intermolecular radical addition and switchable distal sp^3^ C-H bond functionalization from alkyl bromides and vinyl azides. Changing the photocatalyst could chemoselectively control the construction of the C-N bond or C-C bond, which probably resulted from the different pathways in the initial stage, quenching the excited photocatalyst by a reductive or oxidative route. Manipulating the reaction conditions led to different catalytic scenarios and delivered chemodivergent synthesis.

Organic photocatalysts have been extensively explored and utilized in photocatalysis, allowing reactions to occur under metal-free and environmentally benign reaction conditions. Gupta and collaborators (Gupta et al., 2023) have provided a comprehensive summary of the use of organic photocatalysts in the difunctionalization of alkenes and their mechanism pathways, highlighting green and sustainable strategies to construct complex molecules. By using this strategy, Ding et al. developed a difunctionalization of alkenes to provide 3-(arylmethyl)chroman-4-ones via phosphine-mediated C-O bond activation and visible light-mediated C-C bond cleavage. This metal- and oxidant-free reaction system represents a reliable and scalable method to achieve acylation from readily available carboxylic acids. In addition, further functionalization of the products could give rise to diverse core structures which are of great interest in pharmaceutical chemistry.

González et al. contributed with a systematic study of the photophysical properties of depsipeptides and peptoids which were synthesized via a multicomponent reaction. By carefully varying the electron properties of the substituents, different photophysical properties of the products were observed. In addition, some promising photoprotectors and fluorescent probes were discovered in their exploration. Theoretical calculations were in agreement with the experimental UV radiation spectra.

In conclusion, this Research Topic focuses on recent advances in the functionalization of inert or unsaturated bonds via photoredox, and presents fruitful synthetic methodologies and mechanism research achievements in this field. Many interesting discoveries such as Ion-pair effect, chemodivergent synthesis, and hydrogen atom transfer would greatly increase the complexity of the reaction design, and provide more diverse and complex valuable molecules in organic synthesis.

## References

[B1] Bellotti P. Huang H.-M. Faber T. Glorius F. (2023). Photocatalytic late-stage C–H functionalization. Chem. Rev. 123, 4237–4352. 10.1021/acs.chemrev.2c00478 36692361

[B2] Gupta S. Kundu A. Ghosh S. Chakraborty A. Hajra A. (2023). Visible light-induced organophotoredox-catalyzed difunctionalization of alkenes and alkynes. Green Chem. 25, 8459–8493. 10.1039/d3gc03291d

[B3] Holmberg-Douglas N. Nicewicz D. A. (2021). Photoredox-Catalyzed C–H functionalization reactions. Chem. Rev. 122, 1925–2016. 10.1021/acs.chemrev.1c00311 34585909 PMC8939264

[B4] Wang C.-S. Dixneuf P. H. Soulé J.-F. (2018). Photoredox catalysis for building C–C bonds from C(sp2)–H bonds. Chem. Rev. 118, 7532–7585. 10.1021/acs.chemrev.8b00077 30011194

